# An Unusual Case of “Locking” of the Knee Four Years Post Fixation of an Open Supracondylar Femur Fracture

**DOI:** 10.5704/MOJ.1303.005

**Published:** 2013-03

**Authors:** Yuet Peng Khor, Diarmuid Murphy

**Affiliations:** University Orthopaedics, Hand & Reconstructive Microsurgery Cluster, National University Hospital, Singapore; University Orthopaedics, Hand & Reconstructive Microsurgery Cluster, National University Hospital, Singapore

## Abstract

The introduction of locking plate devices have increased the number of viable options for fracture fixation. An understanding of the indications and contraindications and technique limitations are important to minimise associated adverse events. We report the case of a patient who presented with a single broken screw head four years following fixation of a supracondylar femur fracture, resulting in symptoms of migratory knee pain and locking. He underwent implant and foreign body removal and made an unremarkable recovery postoperatively. With the increasing use of locking plate devices, we are likely to see more such complications.

**Key Words:**

bone plates, bone screws, fractures

## Introduction

Locking plates provide improved stability compared to conventional plates 1,2. As screws are locked into the plate, screw failure or pull out rarely occurs unless all adjacent screws fail 1. We present the case of a single broken screw head in a patient 4 years after fixation of a supracondylar femoral fracture with a locking plate that had migrated into the knee joint resulting in knee pain and locking requiring removal of the implant.

## Case Report

**Fig. 1:**
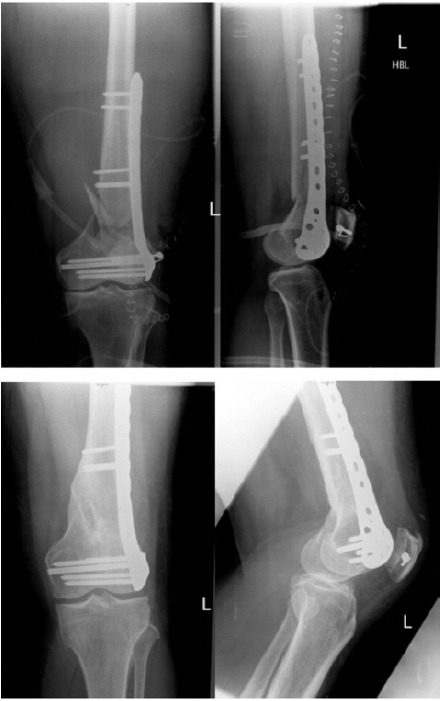
: Immediate post operative anteroposterior and lateral radiographs of the knee (a) and at seven months postoperatively showing complete union (b)

A 42-year-old man was admitted in 2008 following a road traffic accident in which he was a motorcyclist hit by a car. He sustained a Gustillo IIIA AO Type 33C3 left supracondylar femur fracture and a vertical-split fracture of his patella. He underwent surgery for fixation of the supracondylar fracture with a 10 hole Zimmer Periarticular Distal Femoral Locking plate. The patient had an uneventful recovery and his fracture was fully united when reviewed seven months post operatively (Figure 1). He was able to bear full weight with no joint line tenderness, no symptoms of pain or locking of the knee and a range of movement of 0- 135°.

**Fig. 2:**
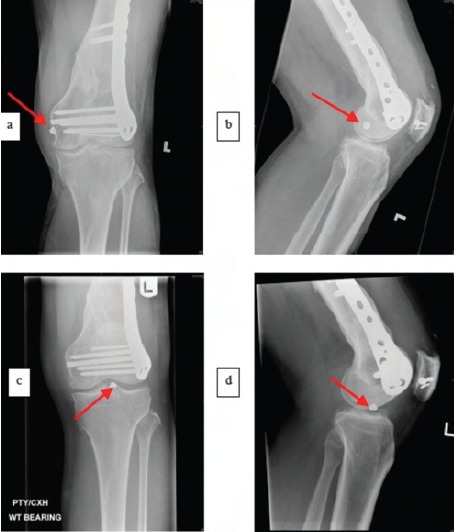
: Radiographs with red arrows indicating the migrated broken screw head on the anteroposterior and lateral radiographs (a, b) of the left knee on initial presentation to the casualty department and its subsequent migration (c, d).

He presented four years later to the casualty department with acute onset of left anteromedial knee pain and locking with no history of trauma. He denied symptoms of pain, locking, fever or erythema during the intervening four years. He had marked discomfort while walking and related how he ‘felt something moving around inside his knee” that occasionally would block his knee movement. On examination, he had no joint line tenderness, McMurray’s test was negative with no ligamentous instability and range of movement of the knee was 5-135°. Plain radiographs revealed a broken screw head on the lateral aspect of the knee. When reviewed in the specialist outpatient clinic a few days later, his symptoms of locking changed to the medial side and repeat radiographs confirmed that the broken screw head had migrated (Figure 2).

**Fig. 3:**
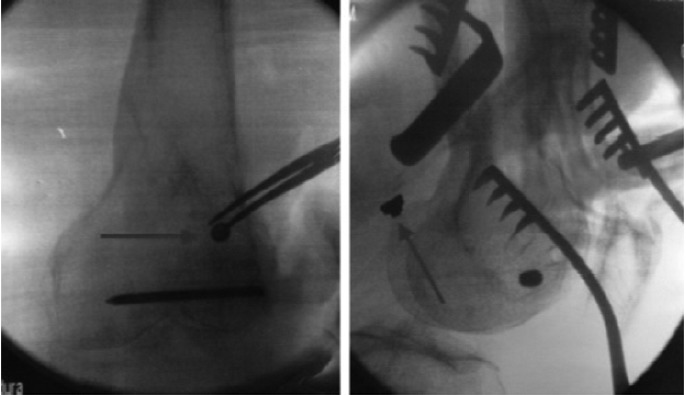
: Intraoperative anteroposterior and lateral fluoroscopic views showing the broken screw head.

The patient underwent removal of foreign body and remaining implants. Intraoperative fluoroscopy confirmed that the broken screw head was lodged on the posterolateral aspect of the femoral condyle (Figure 3); it was removed via the same lateral incision used for removal of the remaining plates and screws. The fracture was fully united and the shaft of the broken screw was left in situ. He had an uncomplicated post-operative recovery.

## Discussion

Cases of broken implants affecting the knee have been reported following anterior cruciate ligament reconstruction, meniscal repair, total knee replacement and fracture fixation. To our knowledge this is the first report of a single broken screw head from a distal femoral locking plate system that resulted in pain and locking of the knee, requiring removal of this loose foreign body.

Locked plate fixation has been used to fix complex periarticular fractures, comminuted metaphyseal or diaphyseal fractures, periprosthetic fractures, and fractures in osteoporotic bone. In the distal femur, locked plate constructs are reported to provide greater fixation strength in response to loading in a cadaveric study performed by Zlowodzki et al. 3. A locked plate device used to repair 103 distal femur fractures resulted in implant failure in the form of loosening in only five cases of a cohort study 4. Fixation failure often occurs due to non union. Repetitive motion in the fracture fragment results in shear stress on metalwork that may lead to implant breakage or failure. In the present case, a single screw head was sheared off although there was no non union or failure of the rest of the implant. Technical reasons for such an occurrence include inadequate insertion and locking of the screw or cross threading within the plate resulting in excessive stress at the head-shaft junction. Unfortunately as the patient did not return for follow up after the initial postoperative visit, we were not able to ascertain the exact time when the screw head broke. The patient however reported that he had remained symptom free until he presented four years later.

The presence of a migrating foreign body in the knee can pose mechanical problems resulting in pain, swelling and locking in addition to damage to the articular surfaces, metallosis and synovitis. Migration of metallic implants to the posterior aspect of the knee may result in serious harm to the neurovascular bundles. For instance, Hsu 5 reported a case of a patellar metal component had migrated to the posterolateral aspect of the knee resulting in knee joint metallosis and a large popliteal cyst. In most reported cases of metal implant migration into the knee, patients underwent arthroscopic procedures for implant removal. In the present case, the patient underwent an open procedure to facilitate the removal of the remainder of the implants.
